# HEALTH PROMOTION A NEW APPROACH TO DEVELOP GERONTOLOGICAL NURSING CARE QUALITY INDICATORS

**DOI:** 10.1093/geroni/igz038.523

**Published:** 2019-11-08

**Authors:** Satomi Kitamura, Ayumi Igarashi, Asa Inagaki, Manami Takaoka, Maiko Noguchi-Watanabe, Mariko Sakka, Takashi Naruse, Noriko Yamamoto-Mitani

**Affiliations:** 1 Department of Gerontological Home Care and Long-term Care Nursing/ Palliative Care Nursing, Division of Health Sciences and Nursing, Graduate School of Medicine, The University of Tokyo, Tokyo, Japan; 2 Department of Community Health Nursing, Division of Health Sciences and Nursing, Graduate School of Medicine, The University of Tokyo, Tokyo, Japan

## Abstract

While the inter-RAI, a comprehensive geriatric assessment tool, contains standardized system for assessing quality of care, there are limitations for its everyday use. Limitations include large number of items, lack of apparent process indicators, and limited symptoms and disease-related information. A new approach was introduced to develop gerontological nursing quality indicators that were targeted at long-term care. We plan to develop staff-friendly indicators, which can be extracted from regular routine client records. A group of nurse researchers discussed essential domains of elderly persons’ life quality, based on nursing theory literature, that nurses strive to maintain. Several outcome indicators were derived out of the domains, and process quality indicators were developed based on literature review. We identified nine domains based on Gordon’s functional health patterns: 1) minimizing symptoms and disease deterioration, 2) maintaining nutritional status, 3) controlling bowel movements, 4) encouraging physical activities, 5) promoting sound sleep, 6) minimizing dementia symptoms, 7) maintaining dignity, and 8) reducing family stress. We then developed 17 outcome indicators; each domain included one to four outcome indicators (e.g., maintaining nutritional status has three indicators: no aspiration, no weight loss by 3% or more, and no dehydration). Process indicators that covered regular assessment (e.g., swallowing evaluation), preventive interventions (e.g., adjusting body positions during a meal), and interventions for the problems (e.g., food texture modification) were determined. These indicators may be useful to assess gerontological nursing quality. We are planning to conduct expert panel and individual client surveys to assess its usability.

